# Utilization of smoking cessation medication benefits among medicaid fee-for-service enrollees 1999–2008

**DOI:** 10.1371/journal.pone.0170381

**Published:** 2017-02-16

**Authors:** Jennifer Kahende, Ann Malarcher, Lucinda England, Lei Zhang, Paul Mowery, Xin Xu, Varadan Sevilimedu, Italia Rolle

**Affiliations:** 1 Centers for Disease Control and Prevention, Atlanta, Georgia, United States of America; 2 Biostatistics Inc., Sarasota, Florida, United States of America; Yokohama City University, JAPAN

## Abstract

**Objective:**

To assess state coverage and utilization of Medicaid smoking cessation medication benefits among fee-for-service enrollees who smoked cigarettes.

**Methods:**

We used the linked National Health Interview Survey (survey years 1995, 1997–2005) and the Medicaid Analytic eXtract files (1999–2008) to assess utilization of smoking cessation medication benefits among 5,982 cigarette smokers aged 18–64 years enrolled in Medicaid fee-for-service whose state Medicaid insurance covered at least one cessation medication. We excluded visits during pregnancy, and those covered by managed care or under dual enrollment (Medicaid and Medicare). Multivariate logistic regression was used to determine correlates of cessation medication benefit utilization among Medicaid fee-for-service enrollees, including measures of drug coverage (comprehensive cessation medication coverage, number of medications in state benefit, varenicline coverage), individual-level demographics at NHIS interview, age at Medicaid enrollment, and state-level cigarette excise taxes, statewide smoke-free laws, and per-capita tobacco control funding.

**Results:**

In 1999, the percent of smokers with ≥1 medication claims was 5.7% in the 30 states that covered at least one Food and Drug Administration (FDA)-approved cessation medication; this increased to 9.9% in 2008 in the 44 states that covered at least one FDA-approved medication (p<0.01). Cessation medication utilization was greater among older individuals (≥ 25 years), females, non-Hispanic whites, and those with higher educational attainment. Comprehensive coverage, the number of smoking cessation medications covered and varenicline coverage were all positively associated with utilization; cigarette excise tax and per-capita tobacco control funding were also positively associated with utilization.

**Conclusions:**

Utilization of medication benefits among fee-for-service Medicaid enrollees increased from 1999–2008 and varied by individual and state-level characteristics. Given that the Affordable Care Act bars state Medicaid programs from excluding any FDA-approved cessation medications from coverage as of January 2014, monitoring Medicaid cessation medication claims may be beneficial for informing efforts to increase utilization and maximize smoking cessation.

## Introduction

Cigarette smoking is the leading cause of premature disease and deaths in the U.S. accounting for approximately 480,000 deaths annually [1]. Medicaid recipients are disproportionately affected by the burden of tobacco use: cigarette smoking prevalence among Medicaid enrollees is significantly higher than in the general adult population (30.1% vs.18.1%) [2]. Smoking accounts for an estimated 15.2% of Medicaid program expenditures, or $40.1 billion annually [1].

Studies suggest that smokers enrolled in Medicaid are as interested in quitting and as likely to make a quit attempt as those in the general population. For example, in 2015, 69.2% of cigarette smokers enrolled in Medicaid indicated they wanted to quit smoking compared with 68.0% in the general population [3]; 56.3% had made a quit attempt for >1 day in the past year compared with 55.4% in the general population [3]; and 5.9% were recent successful quitters compared with 7.4% in the general population. Medicaid-enrolled smokers are also as likely as those in the general population to use cessation treatments [3]. Among current cigarette smokers enrolled in Medicaid who tried to quit in the past year and former smokers who successfully quit in the past 2 years, 32.2% had used a cessation medication and 8.0% used counseling, compared with 29.0% and 6.8%, respectively, of smokers in the general population [3].

Insurance coverage of evidence-based smoking cessation treatments is associated with increased quit attempts, use of cessation treatments, and successful smoking cessation [4]. In 2011, *Healthy People 2020* was established to provide science-based, 10-year national objectives for improving the health of all Americans [5]. One of the *Healthy People 2020* objectives (TU-8) is to increase the number of states whose Medicaid insurance covers all evidence-based smoking cessation treatments including individual, group, and telephone counseling and the seven Food and Drug Administration (FDA)-approved cessation medications [5]. Although progress toward meeting this objective has occurred, many states lack comprehensive cessation coverage. For example, the number of states that covered any counseling increased from 7 in 1999 to 49 in 2014 (however, five of the 49 states restricted counseling to only pregnant women) [6,7,8,9]. Additionally, the number that covered all FDA-approved cessation medications increased from 13 in 1999 to 30 in 2015 [6,7,8,9]. Although the 2010 Affordable Care Act prohibits state Medicaid programs from excluding coverage for FDA-approved smoking cessation medications, including over-the-counter medications, effective January 1, 2014 [10], as of June 30, 2015, based on data from the American Lung Association, 30 state Medicaid programs covered all seven FDA-approved cessation medications [9].

Large state variations have been observed in cessation medication utilization among Medicaid enrollees. For example, a recent study by Ku et al. using Medicaid’s 2013 aggregate state prescription drug utilization rebate files, estimated that utilization of tobacco cessation medication among Medicaid adult smokers varied from 1% in Texas to 27% in Minnesota [11]. To date, few studies have examined the relationship between smoking cessation treatment coverage and cessation outcomes within the Medicaid population. Greene et al. [12] found that enrollees residing in states with the most extensive coverage (counseling and pharmacotherapy with no copayment) had higher rates of successfully quitting in the past 12 months as compared to enrollees residing in states with no coverage and those residing in states in which copayments were required for both counseling and pharmacotherapy. Moreover, Li and Dresler [13] found that the number of tobacco cessation drug claims increased sharply after coverage for nicotine patch and gum started in Arkansas in October 2004, peaked in December 2004, and then declined rapidly; a similar pattern was observed with the addition of varenicline coverage. Their study concluded that Medicaid coverage alone may have a limited, sustained effect on increasing utilization of covered cessation treatments [13]. In addition, Liu [14] observed that smokers residing in states with smoking cessation treatment coverage and no copayment requirements were more likely to have made a quit attempt in the past 12 months and to report intention to quit in the next 6 months than smokers residing in states with no tobacco-dependency treatment coverage. Finally, when Massachusetts widely promoted their new comprehensive cessation benefit coverage to Medicaid enrollees and health care providers, 37% of all enrollees who smoked used the cessation benefit, and cigarette smoking prevalence decreased significantly from 38% in the pre-benefit period to 28% in the post-benefit period [15].

There are currently no studies examining the overall prevalence of utilization of smoking cessation medication benefits among the Medicaid population using individual claims data and how benefit utilization varies by the number or type of medications covered or other individual and state-level characteristics. To fill this gap, we assessed trends in the utilization of smoking cessation medication benefits during 1999–2008 among Medicaid fee-for-service enrollees whose state Medicaid insurance covered at least one smoking cessation medication. We also examined the relationship between cessation medication benefit utilization and coverage of all FDA-approved medications in a given year, the number of medications covered, coverage of varenicline (Chantix) after 2006, individual-level demographic characteristics of Medicaid smokers, and state-level characteristics (cigarette excise tax, statewide smoke-free laws, and per-capita tobacco control program funding).

## Methods

### Data

The Centers for Disease Control and Prevention's National Center for Health Promotion and Disease Prevention’s and its National Center for Health Statistics’ (NCHS) IRB authorities reviewed and approved this study. The study was performed at the NCHS's Research Data Center and no individual level information was released to the study authors. We used data from the 1995 and 1997–2005 National Health Interview Surveys (NHIS) linked to the 1999–2008 Centers for Medicare & Medicaid Services (CMS) Medicaid Analytic eXtract files [16]; no tobacco use questions were included on the NHIS 1996 survey, so subjects interviewed in this year were not included in our analysis. The NHIS utilizes a complex probability sample design to select a nationally representative sample of the civilian, non-institutionalized population of the United States. The household-based survey consists of a core questionnaire with demographic and health-related questions, as well as supplemental questionnaires on a variety of health-related topics.

The Medicaid MAX files include Medicaid enrollment and claims data for NHIS respondents who were successfully matched by the National Center for Health Statistics with Medicaid administrative records. Details of the linkage methodologies and the linked data files are published elsewhere [16].

### Study population

The study population included all NHIS respondents 18–64 years of age during a Medicaid enrollment year who were either current or former cigarette smokers at the time of the NHIS interview and were residents of states whose Medicaid program covered a least one smoking cessation medication at any time from 1999 through 2008. We then limited the population to those who were enrolled in fee-for-service Medicaid for at least one month during the study period, according to the Medicaid claims data. Details on how we obtained our study population are included in Figure A in S1 Appendix.

For our analysis, we examined only periods of enrollment in Medicaid fee-for-service programs, and excluded the data for the entire enrollment year if a person was enrolled in either Medicaid managed care or had dual enrollment in Medicaid and Medicare during any month in a 12-month calendar year. These exclusions were made because for dual enrollees, Medicare is the first payer for services that are covered by both Medicare and Medicaid; MAX only captures these services if additional Medicaid payments are made on behalf of the enrollee for either Medicare cost-sharing or for shared services. In addition, encounter records for those enrollees in Medicaid managed care plans are not provided by all states; in some states, only a portion of managed care recipients have encounter data recorded. Because cessation medication recommendations differ for pregnant women [4] and because charges acquired during pregnancy are often bundled, we also excluded monthly periods of enrollment during pregnancy.

### Cigarette smoking status

Self-reported cigarette smoking information from the NHIS was used to identify respondents as current smokers or former smokers at the time of their interview. Current smokers were defined as those who answered “yes” when asked “Have you smoked at least 100 cigarettes in your entire life?”, and who also answered “every day” or “some days” when asked “Do you now smoke cigarettes every day, some days or not at all?” Former smokers were defined as those who ever smoked ≥ 100 cigarettes, but who answered “not at all” to the aforementioned question. We constructed the person’s past smoking history from NHIS using age of initiation and time since quitting to determine whether they were current smokers at the time of their Medicaid claim. For example, if a person reported that they were a former smoker at time of NHIS interview in 2005, had initiated smoking in 1980 and quit in 2004 then we defined them as a current smoker for any Medicaid claims in the years 1999 (the year the Medicaid claims began) through 2004. For those who were current smokers at the time of their Medicaid claim according to their smoking history, we included Medicaid claims data from years prior to the NHIS interview. For Medicaid claims that occurred after the person’s NHIS interview, we assumed smoking status at the time of the NHIS interview remained constant for the remainder of the study period; only claims for those categorized as ‘current smokers’ at time of Medicaid claim were included.

### Medicaid claims for cigarette smoking cessation medications

The primary outcome measure for this study was whether a current smoker had one or more smoking cessation medication claims in a Medicaid enrollment year. Because enrollees could appear in multiple years, drug claims were assessed for each enrollment year from 1999 to 2008. The percent of Medicaid enrollees with ≥1 cessation medication claim in a given year was calculated. We included all FDA- approved smoking cessation medications (nicotine patch, gum, spray, inhaler, bupropion, lozenges, and varenicline) starting in the year they were approved. Since bupropion is also commonly used for the treatment of depression and other conditions, we also performed sensitivity analyses by excluding bupropion claims from all analysis. Details on drug coding are available in the Text A in S1 Appendix.

### Individual measures

Self-reported gender (male, female), race/ethnicity (Non-Hispanic (NH) White, NH Black, Hispanic, and NH “other”), and educational attainment (< High School, High School/GED, and > High School) of Medicaid enrollees were obtained from the NHIS. Age (18–24, 25–34, 35–44, 45–64 years) was age at Medicaid enrollment year.

### State level measures

Three measures of cessation medication coverage were constructed for each year of the study period: (1) comprehensive drug coverage (i.e., covered all FDA-approved medications; yes/no); (2) number of drugs covered; and (3) varenicline coverage (yes/no). Comprehensive drug coverage was defined as coverage of 5 drugs during 1999–2001, 6 drugs during 2002–2005 (nicotine lozenges were approved in 2002), and 7 drugs during 2006–2008 (varenicline was approved in 2006). Information on state Medicaid drug coverage during 1999–2007 came from published *Morbidity Mortality Weekly Reports* [6,7,8,17,18,19]; data for 2008 were obtained from the American Lung Association [20].

Three state-level variables thought to potentially influence the likelihood of using smoking cessation medications were included in the analysis: 1) state excise taxes on cigarettes (per pack of cigarettes on November 1 of each year [21], adjusted for inflation using 1999 index year); 2) per-capita funding for comprehensive statewide tobacco control programs; and 3) presence or absence of a comprehensive statewide smoke-free law. State funding for tobacco control programs was obtained from the Bridging the Gap/ImpacTeen Project, University of Illinois at Chicago Health Policy Center, [22] where estimates were calculated from the following sources: (1) state appropriations and allocations to tobacco control programs generated from state tobacco excise tax revenues, state settlement payments, and/or state general funds; (2) federal funds received by states and communities for tobacco control and prevention; (3) tobacco control funds from the Robert Wood Johnson Foundation; and (4) tobacco control funds from the American Legacy Foundation. Funding was calculated per capita and was adjusted for inflation using 1999 as the index year [23]. Statewide comprehensive smoke-free laws were determined using the State Tobacco Activities Tracking and Evaluation (STATE) System [23]; states were categorized dichotomously as having a comprehensive smoke-free law covering private workplaces, restaurants and bars versus any other less restrictive policy or no policy. All three state level variables were calculated for each Medicaid enrollment year during 1999–2008.

### Statistical analysis

Three analyses were carried out: 1) descriptive statistics showing the percentage of enrollees who had ≥ 1 claim for a smoking cessation medication in a year, by year and demographic characteristics; 2) time trend tests of the percentages during 1999–2008; and 3) logistic regression to test for independent associations between a Medicaid enrollee’s cessation medication utilization and state Medicaid cessation medication coverage, individual demographic characteristics (age, gender, race/ethnicity, and education), state excise tax, per-capita state tobacco control program funding, and comprehensive state smoke-free law status. The percentage of enrollees with ≥1 medication claim was calculated by summing the indicator variable (whether a person had a cessation medication claim in a given year, 1 = yes, 0 = no) for each enrollment year and dividing that figure by the total number of enrollees who were enrolled for at least one month during that year. Time trends for the annual percentage of enrollees having a claim were carried out using least squares regression and assuming a linear relationship over time. The outcome measure for the logistic models was whether a smoker had one or more smoking cessation medication claims in a Medicaid enrollment year. The three different cessation medication coverage measures were evaluated individually in separate models.

Survey weights were used to account for the probability of selection for each NHIS respondent and a nonresponse adjustment was used to account for demographic differences in linkage eligibility of the linked-MAX files. A post-stratification adjustment was then applied to make the analysis sample demographic distributions match the total MAX file population, by enrollment year. Data were also weighted by the number of enrollment months to control for differences in length of enrollment and eligibility to make a claim. Stratification and cluster variables were included in the analyses to take into account the NHIS multi-stage survey design as well as the fact that respondents could appear in multiple years of the MAX files. SAS® version 9.3 was used for all analyses.

## Results

The number of states that covered at least one drug increased from 30 in 1999 to 45 in 2008, and the number of states that offered comprehensive drug coverage increased from 13 in 1999 (≥5 drugs) to 24 in 2008 (≥7 drugs) (**Table 1**). During 2000–2008, at least 50% of states that covered one or more smoking cessation drugs had comprehensive coverage.

**Table 1:** Number of states whose Medicaid program covered FDA-approved smoking cessation medications,^1^ by number of medications covered and year, 1999–2008.^2^

Number of Medications^1^	1999	2000	2001	2002	2003	2004	2005	2006	2007	2008
**1**	2	2	3	3	4	4	3	0	0	0
**2**	2	2	2	2	1	1	1	2	1	0
**3**	12	11	10	6	8	7	6	3	3	4
**4**	1	1	1	1	1	1	6	2	2	4
**5**	13	16	18	21	20	20	23	5	4	6
**6**	NA	NA	NA	0	0	0	0	8	5	7
**7**	NA	NA	NA	NA	NA	NA	NA	19	26	24
**Total**	30	32	34	33	34	33	39	39	41	45

Abbreviations: NA = Not Applicable

^1^FDA approved nicotine patch, gum, spray in 1996; inhaler and bupropion in 1997; nicotine lozenges in 2002 and varenicline in 2006.

^2^Drug coverage data for 1999–2007 came from published Morbidity Mortality Weekly Reports (MMWR 1998–2000, 1994–2001, 1994–2002, 2005, 2006, 2007) and 2008 data were obtained from the American Lung Association (ALA 2008)[6–8,17–20]. Coverage data from 2002 and 2005 was used to fill in missing information for 2003 and 2004: if there was no change in coverage between 2002 and 2005, we filled in the same information for 2003 and 2004; if 2002 coverage data was different from 2005, we used 2002 data to fill in the missing information for 2003 and 2004, assuming that coverage remained the same until 2005. We excluded the drug coverage if it was for pregnant women-only or for Medicaid Managed Care-only.

The Centers for Medicare and Medicaid Services estimated that those enrolled in fee-for-service represented 59%-61% of the Medicaid population during the midpoint of this study [24]. Among our Medicaid fee-for-service study population, only around one-half were enrolled in Medicaid fee-for-service coverage for the entire year (range: 48.7% in 1999 to 53.0% in 2004; data not shown) and on average they were enrolled for 8.8 months per year (range 8.6 in 1999 to 9.0 in 2003; data not shown). **Table 2** shows demographic characteristics of study participants by whether they had a cessation medication claim. Compared with enrollees without a claim, enrollees with a cessation medication claim were more likely to be in older age groups at the time of Medicaid enrollment (p<0.01). Enrollees with a claim were more likely to be female and to be white compared with those without a claim (74.7% vs. 71.1%; p < .05 and 74.4% vs. 66.2%, respectively; p<0.01).

**Table 2:** Demographic characteristics^1^ of select Medicaid fee-for-service enrollees^2^ who were current cigarette smokers,^3^ by whether they had a smoking cessation medication claim^4^: NHIS (1995, 1997–2005) linked to Medicaid Max files (1999–2008).

		Enrollees with ≥1 cessation medication claim		Enrollees with no cessation medication claim	
Characteristic and Data Source	P-value	Percent	CI	Percent	CI
**Age (years)** (Max)	P<0.01				
18–24		10.0	7.5–12.5	20.5	19.6–21.3
25–34		23.4	20.6–26.2	31.2	30.4–32.0
35–44		20.0	17.7–22.3	18.7	18.2–19.3
45–64		46.7	43.4–49.9	29.6	28.8–30.3
**Gender** (NHIS)	P<0.05				
Male		25.3	22.4–28.1	28.9	28.1–29.6
Female		74.7	71.9–77.6	71.1	70.4–71.9
**Race/Ethnicity** (NHIS)	P<0.01				
White (NH)		74.4	71.6–77.2	66.2	65.4–67.0
Black (NH)		13.8	11.6–16.0	19.8	19.2–20.5
Hispanic		9.5	7.6–11.5	10.7	10.2–11.3
Other (NH)		2.3	1.5–3.1	3.2	2.9–3.6
**Education** (NHIS)	P = 0.31				
Less than HS		42.7	39.5–46.0	42.0	41.1–42.8
HS Diploma /GED		34.2	31.1–37.3	36.6	35.7–37.4
More than HS		23.1	20.3–25.8	21.5	20.8–22.2

Abbreviations: NH = Non Hispanic; HS = High school; GED = General Education Development; CI = confidence Interval

P-value from test of association between the domain and a variable measuring whether or not the enrollee had a claim.

^1^Information on demographic (except age) and smoking characteristics are from the National Health Interview Surveys (NHIS) conducted in 1995, 1997–2005. Age was recoded as age at Medicaid enrollment.

^2^Includes persons aged 18–65 years in the NHIS/MAX linked file from states that covered at least one cessation medication from 1999–2008. Only persons who were enrolled in fee-for-service Medicaid for at least one month are included.

Excludes monthly periods of enrollment for women enrolled due to pregnancy. Excludes the monthly data for an entire enrollment year if a person during any month in a 12-month calendar year was enrolled in either Medicaid managed care or had dual enrollment in Medicaid and Medicare.

^3^Current smokers at the time of their NHIS interview. For current smokers (persons who ever smoked 100 cigarettes and who reported smoking every day or some days) at NHIS interview we used data on age of initiation to capture all claims since they started smoking. For former smokers (persons who ever smoked 100 cigarettes and did not smoke at time of the interview),we used time since quitting to capture all claims when they were a current smoker. For claims after time of NHIS interview we assumed smoking status at NHIS remained constant for the remainder of the study.

^4^U.S. Food and Drug Administration approved cessation medications were nicotine patch, gum, spray, inhaler, bupropion, lozenges and varenicline.

**Table 3** shows the percentage of smokers enrolled in Medicaid fee-for-service with one or more smoking cessation medication claims. In 1999, 5.7% of smokers had one or more claims, which increased to 9.9% in 2008 (p<0.01). Among those with at least one cessation medication claim in a given year, the percentage with ≥2 claims ranged from 43.6% in 2006 to 60.9% in 2001 (data not shown). Percentages generally increased for all age groups, both genders, and for all three racial/ethnic and education groups.

**Table 3:** Percentage^1^ of select Medicaid fee-for-service enrollees who were current cigarettesmokers^1^ in fee-for-service who had one or more cessation medication claims by year^2, 3, 4^: NHIS (1995, 1997–2005) linked to Medicaid Max files (1999–2008).

	Medicaid Enrollment Year										
	1999	2000	2001	2002	2003	2004	2005	2006	2007	2008	p-value 1999–2008
**Characteristic and Data Source**											
**Total**	5.7	5.4	6.5	6.2	7.3	5.0	3.7	5.9	9.7	9.9	p<0.01
**Age (years)**^**5**^ (Max)											
25–34	5.5	3.5	5.6	5.3	5.7	3.5	2.9	2.4	7.3	7.1	p = 0.49
35–44	4.5	6.1	7.5	9.5	6.8	4.5	3.3	6.7	9.8	10.7	p = 0.10
45–64	7.8	9.6	10.7	8.2	9.4	6.8	5.1	9.8	12.9	12.8	p = 0.06
**Gender** (NHIS)											
Male	5.3	3.7	7.4	5.5	5.8	5.0	2.4	6.7	8.6	8.2	p = 0.14
Female	5.8	6.0	6.2	6.5	8.0	5.0	4.2	5.5	10.0	10.5	p<0.01
**Race/Ethnicity**^**6**^ ((NHIS)											
White (NH)	7.1	6.0	8.2	7.0	8.6	5.2	3.7	6.5	10.2	11.6	p<0.05
Black (NH)	2.9	4.4	2.9	3.7	4.8	3.6	2.3	5.2	7.8	6.6	p<0.05
Hispanic	3.2	4.4	5.1	5.9	4.6	5.6	7.0	3.8	11.0	6.9	p<0.05
**Education** (NHIS)											
Less than HS	4.2	5.8	6.5	6.2	7.4	5.0	4.3	6.3	10.7	8.6	p<0.05
HS Diploma /GED	6.6	4.8	5.8	5.4	6.6	4.9	2.6	5.8	8.2	10.9	p = 0.06
More than HS	7.2	5.6	7.8	7.7	8.0	5.1	4.3	5.2	8.3	10.4	p = 0.49

Abbreviations: NH = Non Hispanic; HS = High school; GED = General Education Development

^1^Current smokers at the time of their NHIS interview. For current smokers (persons who ever smoked 100 cigarettes and who reported smoking every day or some days) at NHIS interview we used data on age of initiation to capture all claims since they started smoking. For former smokers (persons who ever smoked 100 cigarettes and did not smoke at time of the interview), we used time since quitting to capture all claims when they were a current smoker. For claims after time of NHIS interview we assumed smoking status at NHIS remained constant for the remainder of the study.

^2^Smoking cessation medication utilization is defined as the percentage of current cigarette smokers in fee-for-service Medicaid with at least one smoking cessation medication claim. This percentage was weighted by person-months of enrollment calculated separately for each person and enrollment year. The same enrollee may appear in multiple years.

^3^Smoking cessation medications: nicotine patch, gum, spray, inhaler, bupropion, lozenges and varenicline.

^4^Includes persons aged 18–65 years in the NHIS/MAX linked file from states that covered at least one cessation medication from 1999–2008. Only persons who were enrolled in fee-for-service Medicaid for at least one month are included. Excludes monthly periods of enrollment for pregnant women enrolled due to pregnancy. Excludes the monthly data for an entire enrollment year if a person during any month in a 12-month calendar year was enrolled in either Medicaid managed care or had dual enrollment in Medicaid and Medicare.

^5, 6^ The “18–24” category for age and “other” level for race were not reportable due to RSE > 30%.

### Multivariate analysis

Comprehensive drug coverage (AOR = 1.6; 95% CI: 1.3–2.0; p < .05) (model 1), covering 5 or more cessation medications (AOR = 2.4; 95% CI: 1.4–5.0; p < .05) (model 2), and varenicline coverage (AOR = 4.2; 95% CI: 2.8–6.4; p <0.05) (model 3) were all significantly associated with having a cessation medication claim (**Table 4**). The relationships between the individual demographic characteristics and cessation medication utilization that were observed in the bivariate analyses remained significant in all three multivariate models. State excise tax and per-capita tobacco control funding were positively associated with having a claim. Comprehensive statewide smoke-free laws were not associated with having a medication claim.

**Table 4:** Multivariate logistic regression models for correlates of having a claim for smoking cessation medication,^1^ among current smokers^2^ who were enrolled in Medicaid Fee-for-Service^3^^,^^4^, Source: NHIS (1995, 1997–2005) linked to Max files, 1999–2008.

Characteristic and Data Source	Model 1 Comprehensive cessation edication coverage^5^		Model 2 Number of cessation medications in state benefit		Model 3 Coverage ofvarenicline^6^	
	OR	(95% CI)	OR	(95% CI)	OR	(95% CI)
**Comprehensive Drug Coverage**^**5**^						
No	Ref		**-**	**-**	**-**	**-**
Yes	**1.62**	(1.31–2.01)	**-**	**-**	**-**	**-**
**Number of cessation drugs in statebenefit**^**5**^						
1	**-**	**-**	Ref		**-**	**-**
2	**-**	**-**	1.92	(0.72–5.08)	**-**	**-**
3	**-**	**-**	1.50	(0.70–3.22)	**-**	**-**
4	**-**	**-**	2.21	(0.96–5.06)	**-**	**-**
5 or more	**-**	**-**	**2.38**	(1.41–4.95)	**-**	**-**
**Varenicline Coverage**^**6**^						
No	**-**	**-**	**-**	**-**	Ref	
Yes	**-**	**-**	**-**	**-**	**4.25**	(2.81–6.43)
**Age (years) (Max)**						
18–24	Ref		Ref		Ref	
25–34	1.13	(0.73, 1.74)	1.12	(0.72, 1.74)	1.33	(0.55, 3.21)
35–44	**1.90**	(1.25, 2.87)	**1.84**	(1.21, 2.79)	**2.51**	(1.01, 6.21)
45–64	**2.60**	(1.72, 3.94)	**2.50**	(1.64, 3.81)	**3.29**	(1.29, 8.38)
**Gender (NHIS)**						
Male	Ref		Ref		Ref	
Female	**1.60**	(1.31, 1.96)	**1.63**	(1.33, 1.99)	**1.52**	(1.11, 2.09)
**Race/Ethnicity (NHIS)**						
Black (NH)	Ref		Ref		Ref	
White (NH)	**1.71**	(1.33, 2.20)	**1.72**	(1.34, 2.21)	**1.63**	(1.09, 2.45)
Hispanic	1.19	(0.87, 1.63)	1.16	(0.85, 1.59)	0.83	(0.51, 1.38)
Other, (NH)	1.01	(0.54, 1.88)	1.08	(0.58, 2.02)	0.68	(0.28, 1.64)
**Education (NHIS)**						
More than HS	Ref		Ref		Ref	
HS Diploma /GED	0.96	(0.73, 1.26)	0.94	(0.72, 1.23)	1.22	(0.78, 1.92)
Less than HS	1.14	(0.88, 1.48)	1.11	(0.86, 1.43)	**1.49**	(1.02, 2.17)
**State Excise Tax**^**7**^^,^*	**1.18**	(1.08, 1.29)	**1.14**	(1.04, 1.26)	**1.16**	(1.02, 1.31)
**State Funding Per Capita**^**8**^^,^*	**1.05**	(1.02, 1.08)	**1.04**	(0.99, 1.80)	**1.07**	(1.01, 1.12)
**State Comprehensive Smoke-free law**^**9**^						
No	Ref		Ref		Ref	
Yes	1.33	(0.99, 1.80)	1.34	(1.00, 1.07)	1.32	(0.93, 1.88)

*Continuous variable Blank cell means estimates not applicable to model. P-value for the Wald Chi-squared was <0.05 (bolded in table).

^1^The dependent variable was defined as having at least one smoking cessation medication claim in an enrollment year. The same enrollee may appear in multiple years.

^2^Current smokers at the time of their NHIS interview. For current smokers (persons who ever smoked 100 cigarettes and who reported smoking every day or some days) at NHIS interview we used data on age of initiation to capture all claims since they started smoking. For former smokers (persons who ever smoked 100 cigarettes and did not smoke at time of the interview), we used time since quitting to capture all claims when they were a current smoker. For claims after time of NHIS interview we assumed smoking status at NHIS remained constant for the remainder of the study.

^3^Includes persons aged 18–65 years in the NHIS/MAX linked file from states that covered at least one cessation medication from 1999–2008. Only persons who were enrolled in fee-for-service Medicaid for at least one month are included. Excludes monthly periods of enrollment for women enrolled due to pregnancy. Excludes the monthly data for an entire enrollment year if a person during any month in a 12-month calendar year was enrolled in either Medicaid managed care or had dual enrollment in Medicaid and Medicare.

^4^Claims were weighted by person-months of enrollment.

^5^Comprehensive drug coverage was defined as coverage of 5 drugs in 1999–2001, 6 drugs in 2002–2005, and 7 drugs in 2006–2008 (source: references 6–8, 17–20).

^6^This model is limited to 2006–2008 in that varenicline was approved by the FDA in 2006. Coverage of varenicline was defined as living in a state with Medicaid coverage of varenicline (source: references 18–20).

^7^State excise tax was defined as total state excise taxes per pack adjusted for inflation for the enrollee’s state and year (source: Orzechowski and Walker (2012).

^8^State funding per capita was defined as amount in dollars of funds per person for the enrollee’s state and year available for tobacco control. Includes funding from federal sources and private foundations. Source: Bridging the Gap/ImpacTeen Project, University of Illinois at Chicago Health Policy Center (http://www.bridgingthegapresearch.org/).

^9^State comprehensive smoke-free law was defined as a law prohibiting smoking in all indoor areas of private worksites, restaurants, and bars in the enrollee’s state and year (source: CDC STATE system).

In our sensitivity analyses where bupropion claims excluded, we observed similar trends over time in medication utilization as well as similar relationships between the individual- and state-level characteristics and medication claims.

## Discussion

During 1999–2008, both the number of states that covered at least one FDA-approved smoking cessation medication and the number of states with comprehensive cessation medication coverage increased. In addition, among smokers from states whose Medicaid program covered at least one medication and who were enrolled in Medicaid fee-for-service, the percentage with one or more cessation medication claims also increased from 5.7% to 9.9% during the same time period. Comprehensive coverage, the number of cessation medications covered, and varenicline coverage, which was approved by the FDA in 2006, were all positively associated with having a medication claim. Cessation medication claims also varied by the enrollee’s demographic characteristics and the state’s excise tax and per-capita tobacco control funding. While it is encouraging that utilization of cessation medication is increasing among Medicaid enrollees who smoke, use remains low. The 9.9% utilization in our study is similar to the 10.0% utilization found in 2013 by Ku et al. [11] using 2010–2013 Medicaid state aggregate prescription drug rebate data that included both fee-for-service and managed care prescriptions. Well-funded comprehensive state tobacco control programs which include anti-tobacco mass media campaigns, increased cigarette excise taxes, and barrier-free access to cessation treatments are needed to further increase cessation among the smoking population in general including Medicaid enrollees [4,25,26].

Our observation of increased utilization of cessation medications with increased coverage underscores the importance of offering comprehensive cessation benefits to Medicaid smokers. Use of FDA-approved cessation medications in combination with counseling by individuals can double or triple quit rates [4]. The 2008 update to the Public Health Service Clinical Practice Guideline on Treating Tobacco Use and Dependence [4] determined that providing tobacco dependence treatments (both medication and counseling) as a paid or covered benefit by health insurance plans increases the proportion of smokers who use cessation treatment, attempt to quit, and successfully quit. Additionally, the guideline recommends that cessation treatments be included as covered services in public and private health benefit plans [4]. Although coverage of medications increased during 1999–2008, as of June 2015, only nine states met the Healthy People TU-8 objective [5] of Medicaid programs covering individual, group, and telephone counseling and all seven FDA-approved cessation medications [9,25]. Accordingly, it is critical that state tobacco control programs continue to work with their Medicaid programs to expand coverage, remove barriers to coverage utilization by eliminating co-pays and prior authorization, and promote cessation treatment coverage to enrollees and providers [9, 25].

Consistent with what we found with varenicline coverage, Li and Dresler [13] observed increased utilization following the addition of varenicline coverage among Arkansas Medicaid enrollees. In 2006, 32 states added varenicline coverage, which likely caused an increase in utilization that could be detected at an aggregate level in our study.

We also observed differences in utilization by demographic characteristics of the Medicaid enrollees. Older age, female gender, and white/non-Hispanic race/ethnicity were positively associated with cessation medication claims. These finding are consistent with those of Li and Dresler [13], who also found that age ≥ 25 years and white, non-Hispanic race/ethnicity were positively associated with utilization of medication benefits in Arkansas. These demographic patterns are also consistent with national data in which self-reported use of medication during smokers’ last quit attempt was associated with being female, older, and non-Hispanic white [3,27,28]. The demographic differences in medication utilization may reflect patterns of health care utilization, receipt of cessation advice from a health care professional, and knowledge, attitudes, and beliefs related to the effectiveness and safety of tobacco cessation medications. Women and non-Hispanic whites are more likely to utilize health care than men and other racial/ethnic groups, respectively, and utilization of health care also increases with age [29]. Among those who have seen a health care provider, receipt of counseling increases with age [3,30]. Finally, studies show that there is a lack of knowledge about nicotine replacement therapy effects and its efficacy, as well as the benefits of pharmacotherapy for cessation in general across racial and ethnic groups [31–35]. It is critical that advice to quit and assistance with cessation, including discussing the benefits of cessation medications, be consistently offered to all patients [4].

State excise tax and state per capita funding for tobacco control programs were positively associated with having a Medicaid claim for a smoking cessation medication. These findings are consistent with prior literature that found that increases in cigarette tax are positively associated with quit attempts, successful quits [25, 36–39], and the use of quitline counseling and nicotine replacement therapy [40–41]. Increased funding for state tobacco control programs is associated with the ability of states to educate consumers about the dangers of tobacco products and thereby also motivates quit attempts and use of quitlines [25, 38, 42–43]. Although comprehensive statewide smoke-free laws were not associated with cessation medication utilization in this study they have been found to increase population-level rates of smoking cessation [26].

The strength of this study is that it uses individual Medicaid claims data across multiple states over time and links smoking behavior to medical claims; however, the study has some limitations. We limited claims to those from fee-for-service plans due to the incomplete claims data for those enrolled in Medicaid managed care plans and our results are not generalizable to the entire Medicaid enrolled population. Also, only those Medicaid fee-for-service enrollees in the MAX file who were found in the NHIS, and thus eligible to be linked, were included in the analysis. A secondary analysis comparing the linked NHIS-MAX file population to the NHIS Medicaid population revealed that the linked file population was, on average, more likely to be younger, female, non-Hispanic white, and to have a high-school diploma or GED. Since many of these populations are more likely to use cessation treatments than other demographic groups [3, 27–28], we may have overestimated the utilization rate of Medicaid cessation benefits. It is important to note that the post-stratification of the sample weights to administrative data totals and the effect of combining the samples and re-weighting the annual national weights used in this analysis has not been well studied. We made several assumptions regarding the weights, for example, that the post-stratification cells were reasonably homogenous even given that the participants were from different NHIS survey years. Prevalence estimates may be particularly affected by this choice of sample weighting and should be interpreted with caution. In addition, although the same NHIS respondent could appear in the analysis dataset for more than one Medicaid enrollment year, our intent was not to model the pattern of cessation claims for individuals over time. Rather we estimated the population average cessation drug claim rates by year. For analyses that included data from multiple enrollment years (for example, see Table 4), we accounted for any variance inflation due to the same person in multiple years by modeling the covariance structure of the dataset. This covariance structure was included in the estimation algorithm. Although respondents who appeared in most or all Medicaid enrollment years could potentially bias the results, the proportion of NHIS respondents who appeared in more than one year is small and probably not a source of bias.

Some of the measures also have limitations. Cigarette smoking status was determined at the time of the NHIS interview, and we assumed that all smokers continued to smoke throughout the study period. It is likely that some study participants quit after the NHIS interview, in that approximately 7% of cigarette smokers quit in a given year [3]. There is also a possibility that some enrollees may have started smoking after age 18 years [44] and some of those who were formers smokers at the time of NHIS interview may have relapsed to smoking [45]. Similarly, education was determined at NHIS interview and may have changed over time particularly for people who were interviewed prior to age 25. We also may have included cessation medication utilization claims from persons who were using bupropion for treatment of depression or other conditions, and not smoking cessation. However, when we excluded bupropion claims from the analysis, our trend and logistic models results did not change. Finally, we were not able to assess whether the enrollees received advice to quit, smoking cessation counseling or important factors that affect medication utilization (e.g., awareness/promotion of benefits or potential barriers to accessing treatments). We did not analyze data on smoking cessation counseling due to small sample sizes (< 1% of current smokers had a Medicaid counseling claim). In addition, advice to quit was not examined because that question was only included in the 2000 and 2005 NHIS and state-level data on advice to quit and health insurance from the Behavioral Risk Factor Surveillance System was available for only the year 2000 for 19 states (http://www.cdc.gov/brfss/questionnaires/index.htm). The combined use of cessation counseling and cessation medications is more effective in increasing quit rates than the use of either alone [4].

In January 2014, the Affordable Care Act barred states programs from excluding FDA-approved cessation medications from coverage [10]. This expansion of coverage should hopefully accelerate the increasing trends of cessation medication utilization by Medicaid enrollees who smoke observed in this study. However, the impact of the provision will depend on state implementation, and optimal utilization of treatments will most likely occur in states that add cessation medications to their Medicaid preferred drug lists and remove other barriers to accessing these medications [9]. Although expansion of Medicaid coverage of effective cessation treatments will increase their accessibility, it will not guarantee sustained rates of increased utilization [13, 42–43, 46]. Therefore, efforts are warranted to increase the adoption of comprehensive tobacco control programs that work to motivate tobacco users to quit [26, 37], particularly given that this study found that levels of both state tobacco control program funding and state cigarette excise taxes were independently associated with increased rates of cessation medication utilization among Medicaid enrollees who smoke. Population-based strategies that increase cessation are particularly important given that the majority of cigarette smokers now quit without using behavioral interventions or medication [3]. Targeted promotions are also critical to educate Medicaid enrollees and their health care providers about Medicaid’s cessation treatment coverage and the effectiveness of cessation treatments. Health care providers are missing many opportunities to provide cessation advice, counseling, and medications to patients. For example, estimates from the 2015 National Health Interview Survey show that 89.8% of smokers aged 18–64 years with Medicaid saw a doctor or dentist in the past year. However, among those who saw a doctor or dentist 40.0% (2.7 million) did not receive advice to quit and among those who were advised to quit 63.3% (2.3 million) did not use any effective treatment for their last quit attempt (https://www.cdc.gov/nchs/nhis/nhis_2015_data_release.htm). Implementation of tobacco policies and programs that motivate tobacco users to quit, expand comprehensive Medicaid coverage of cessation treatments, increase awareness of Medicaid cessation benefits, educate on the effectiveness of cessation treatments, and institutionalize health systems changes that make tobacco use screening and treatment the standard of care have the potential to increase utilization of effective cessation treatments and long-term cessation among Medicaid enrollees [4, 25–26, 37].

## Supporting information

S1 Appendix Sample Flow Chart (Figure A). Drug Code (Text A).
(DOCX)

## References

[1] US Department of Health and Human Services. The Health Consequences of Smoking—50 Years of Progress: A Report of the Surgeon General. Atlanta, GA: US Department of Health and Human Services, Centers for Disease Control and Prevention, National Center for Chronic Disease Prevention and Health Promotion, Office on Smoking and Health 2014.

[2] Blackwell DL, Lucas JW, Clarke TC. Summary Health Statistics for U.S. Adults: National Health Interview Survey, 2012. Vital Health Stat. 2014;10: 1–161. 24819891

[3] Centers for Disease Control and Prevention. Quitting Smoking among Adults—United States, 2000–2015. MMWR. 2017; 65;1457–1464. 10.15585/mmwr.mm6552a1 28056007

[4] Fiore MC, Jaén CR, Baker TB, Bailey WC, Benowitz NL, Curry SJ, et al Treating Tobacco Use and Dependence: 2008 Update. Clinical Practice Guideline. Rockville, MD: U.S. Department of Health and Human Services. Public Health Service 5 2008.

[5] United States Department of Health and Human Services. Healthy People 2020. Washington, DC. 2011. Available from: http://www.healthypeople.gov/2020/topicsobjectives2020/pdfs/HP2020objectives.pdf.

[6] Centers for Disease Control and Prevention 4. State Medicaid Coverage for Tobacco-Dependence Treatments—United States, 1998 and 2000. MMWR. 2001;50: 979–982. 11724152

[7] Centers for Disease Control and Prevention. State Medicaid Coverage for Tobacco-Dependence Treatments—United States, 1994–2001. MMWR. 2003;52: 496–500. 12809111

[8] Centers for Disease Control and Prevention. State Medicaid Coverage for Tobacco-Dependence Treatments—United States, 1994–2002. MMWR. 2004;53: 54–57. 14749613

[9] Centers for Disease Control and Prevention. State Medicaid Coverage for Tobacco Cessation Treatments and Barriers to Coverage—United States, 2014–2015. MMWR. 2015;64: 1194–9. 10.15585/mmwr.mm6442a3 26513425

[10] Patient Protection and Affordable Care Act 2010. Public Law 111–148. Title IV, §4207, USC HR 3590, 2010.

[11] Ku L, Bruen BK, Steinmetz E, Bysshe T. Medicaid Tobacco Cessation: Big Gaps Remain in Efforts To Get Smokers To Quit. Health Aff. 2016;35:62–70. 10.1377/hlthaff.2015.0756 PMC4705858 26733702

[12] Greene J, Sacks RM, McMenamin SB. The Impact of Tobacco Dependence Treatment Coverage and Copayments in Medicaid. Am J Prev Med. 2014;46: 331–336. 10.1016/j.amepre.2013.11.019 24650835

[13] Li CH, Dresler CM, Medicaid Coverage and Utilization of Covered Tobacco-Cessation Treatments the Arkansas Experience. Am J Prev Med. 2012;42: 588–595. 10.1016/j.amepre.2012.02.018 22608374

[14] Liu F. Quit Attempts and Intention to Quit Cigarette Smoking among Medicaid Recipients in the USA. Public Health. 2010;124: 553–558. 10.1016/j.puhe.2010.05.015 20832833

[15] Land T, Warner D, Paskowsky M, Cammaerts A, Wetherell L, Kaufmann R, et al Medicaid Coverage for Tobacco Dependence Treatments in Massachusetts and Associated Decreases in Smoking Prevalence. PLoS One. 2010 3 18;5(3):e9770 10.1371/journal.pone.0009770 20305787 PMC2841201

[16] Simon AE, Driscoll AK, Golden C, Tandon R, Duran CR, Miller EA, et al Documentation and Analytic Guidelines for NCHS Surveys Linked to Medicaid Analytic Extract (Max) Files. Hyattsville, MD: National Center for Health Statistics 2014 Available from: http://www.cdc.gov/nchs/data/datalinkage/documentation_and_analytic_guidelines_nchs_survey_max_linked_data.pdf.

[17] Centers for Disease Control and Prevention. State Medicaid Coverage for Tobacco-Dependence Treatments—United States, 2005. MMWR. 2006;55: 1194–1197. 17093384

[18] Centers for Disease Control and Prevention. State Medicaid Coverage for Tobacco-Dependence Treatments—United States, 2006. MMWR. 2008;57: 117–122. 18256583

[19] Centers for Disease Control and Prevention. State Medicaid Coverage for Tobacco-Dependence Treatments—United States, 2007. MMWR. 2009;58: 1199–1204. 19893479

[20] American Lung Association. Tobacco Policy Trend Report: Helping Smokers Quit 2008. 2008. Available from: http://www.lung.org/assets/documents/publications/smoking-cessation/helping-smokers-quit2008.pdf.

[21] Orzechowski W, Walker R. The Tax Burden on Tobacco. Historical Compilation. Arlington, VA: Orzechowski and Walker Consulting 2007.

[22] Bridging the Gap/ImpacTeen Project, University of Illinois at Chicago Health Policy Center. Available from: http://www.bridgingthegapresearch.org/.

[23] Centers for Disease Control and Prevention State Tobacco Activities Tracking and Evaluation (State) System. Available from: http://www.cdc.gov/tobacco/statesystem.

[24] Centers for Medicare and Medicaid Services. Medicaid Managed Care Enrollment Report. Summary statistics as of July 1, 2001. 2014. Available from: http://www.medicaid.gov/Medicaid-CHIP-Program-Information/By-Topics/Data-and-Systems/Downloads/2011-Medicaid-MC-Enrollment-Report.pdf.

[25] Centers for Disease Control and Prevention. Best Practices for Comprehensive Tobacco Control Programs—2014. Atlanta: U.S. Department of Health and Human Services, Centers for Disease Control and Prevention, National Center for Chronic Disease Prevention and Health Promotion, Office on Smoking and Health. 2014.

[26] Guide to Community Preventive Services. Reducing Tobacco Use and Secondhand Smoke Exposure: Interventions to Increase the Unit Price for Tobacco Products. 2012. Available from: www.thecommunityguide.org/tobacco/increasingunitprice.html.

[27] Shiffman S, Brockwell SE, Pillitteri JL, Gitchell JG. Individual Differences in Adoption of Treatment for Smoking Cessation: Demographic and Smoking History Characteristics. Drug Alcohol Depend. 2008;93: 121–131. 10.1016/j.drugalcdep.2007.09.005 17996399

[28] Curry SJ, Sporer AK, Pugach O, Campbell RT, Emery S. Use of Tobacco Cessation Treatments among Young Adult Smokers: 2005 National Health Interview Survey. Am J Public Health. 2007;97: 1464–1469. 10.2105/AJPH.2006.103788 17600243 PMC1931476

[29] O’Hara B, Caswell K. Health Status, Health Insurance, and Medical Services Utilization: 2010. Curr Pop Rep. 2012; 70–133.

[30] Frieden TR. Use of Selected Clinical Preventive Services Among Adults—United States, 2007–2010. MMWR. 2012;61 Suppl: 1–2. 22695456

[31] Carpenter MJ, Ford ME, Cartmell K, Alberg AJ. Misperceptions of Nicotine Replacement Therapy within Racially and Ethnically Diverse Smokers. J Natl Med Assoc 2011;103: 885–894. 22364057 10.1016/s0027-9684(15)30444-2 PMC3372924

[32] McMenamin SB, Halpin HA, Bellows NM. Knowledge of Medicaid Coverage and Effectiveness of Smoking Treatments. Am J Prev Med. 2006;31: 369–374. 10.1016/j.amepre.2006.07.015 17046407

[33] Levinson AH, Perez-Stable EJ, Espinoza P, Flores ET, Byers TE. Latinos Report Less Use of Pharmaceutical Aids When Trying to Quit Smoking. Am J Prev Med. 2004;26: 105–111. 14751320 10.1016/j.amepre.2003.10.012

[34] Fu SS, Burgess D, van Ryn M, Hatsukami DK, Solomon J, Joseph AM. Views on Smoking Cessation Methods in Ethnic Minority Communities: a Qualitative Investigation. Prev Med. 2007;44: 235–240. 10.1016/j.ypmed.2006.11.002 17175016

[35] Cook-Shimanek M, Burns EK, Levinson AH. Medicinal Nicotine Nonuse: Smokers' Rationales for Past Behavior and Intentions to Try Medicinal Nicotine in a Future Quit Attempt. Nicotine Tob Res. 2013;15: 1926–1933. 10.1093/ntr/ntt085 23817584 PMC3842129

[36] Chaloupka FJ, Straif K, Leon ME. Effectiveness of Tax and Price Policies in Tobacco Control. Tob Control. 2011;20: 235–238. 10.1136/tc.2010.039982 21115556

[37] Chaloupka FJ, Warner KE. The Economics of Smoking In: Anthony JC, Joseph PN, editors. Handbook of Health Economics: Elsevier 2000 pp. 1539–1627.

[38] Community Preventive Service Task Force. The Guide to Community Preventive Services: Tobacco Recommendations. Available from: https://www.thecommunityguide.org/topic/tobacco.

[39] Wilson LM, Avila Tang E, Chander G, Hutton HE, Odelola OA, Elf JL, et al Impact of Tobacco Control Interventions on Smoking Initiation, Cessation, and Prevalence: A Systematic Review. J Environ Public Health 2012;2012:961724 10.1155/2012/961724 22719777 PMC3376479

[40] Bush T, Zbikowski S, Mahoney L, Deprey M, Mowery PD, Magnusson B. (2012) The 2009 US Federal Cigarette Tax Increase and Quitline Utilization in 16 States. J Environ Public Health 2012: 314740 2012;2012:314740. 10.1155/2012/314740 22649463 PMC3356941

[41] Metzger KB, Mostashari F, Kerker BD. Use of Pharmacy Data to Evaluate Smoking Regulations' Impact on Sales of Nicotine Replacement Therapies in New York City. Am J Public Health. 2005;95: 1050–1055. 10.2105/AJPH.2004.048025 15914832 PMC1449307

[42] Kotz D, Fidler J, West R. Factors Associated with the Use of Aids to Cessation in English Smokers. Addiction. 2009;104: 1403–1410. 10.1111/j.1360-0443.2009.02639.x 19549267

[43] Bondy SJ, Diemert LM, Victor JC, McDonald PW, Cohen JE. Assessing the Reach of Nicotine Replacement Therapy as a Preventive Public Health Measure. Chronic Dis Inj Can 2012;33: 19–28. 23294918

[44] U.S. Department of Health and Human Services. Preventing Tobacco Use Among Youth and Young Adults: A Report of the Surgeon General. Atlanta, GA: U.S. Department of Health and Human Services, Centers for Disease Control and Prevention, National Center for Chronic Disease Prevention and Health Promotion, Office on Smoking and Health, 2012.

[45] Hughes JR, Keely J, Naud S. Shape of the relapse curve and long-term abstinence among untreated smokers. Addiction. 2004;99: 29–38. 14678060 10.1111/j.1360-0443.2004.00540.x

[46] Fu SS, Sherman SE, Yano EM, van Ryn M, Lanto AB, Joseph AM. Ethnic Disparities in the Use of Nicotine Replacement Therapy for Smoking Cessation in an Equal Access Health Care System. Am J Health Promot. 2005;20: 108–116. 16295702 10.4278/0890-1171-20.2.108

