# Supplementary material for: Utilization of smoking cessation medication benefits among medicaid fee-for-service enrollees 1999–2008
Source: PLoS One. 2017 Feb 16;12(2):e0170381. doi: 10.1371/journal.pone.0170381 (PMC5313220; doi:10.1371/journal.pone.0170381)
Supplement: S1 Appendix — Sample Flow Chart (Figure A). Drug Code (Text A). (DOCX) [file pone.0170381.s001.docx]

**Appendix:**

**A. Sample flow chart**

Claims for smoking cessation medications among Medicaid enrollees who were smokers were examined in the linked NHIS/MAX data file. Linkage of NHIS survey respondents to CMS administrative records is performed for those NHIS respondents who are “linkage-eligible”. Linkage-eligibility is determined by whether NHIS respondents provided the necessary information: Social Security number (SSN) and date of birth. The linked file is smaller than the original sample of NHIS respondents because (1) some NHIS respondents were not linkage-eligible, and (2) many who were linkage-eligible were not enrolled in Medicaid during our study period.

NHIS Sample Adult files 1995, 1997 – 2005

**N=307,024**

Retain only current smokers, for each enrollment year*

NHIS Sample Adult file respondents eligible for match to MAX Person Summary (PS) file

**N=174,302**

*Current smoking status at each enrollment year was determined by the enrollee’s responses to NHIS items asking about current smoking status, year started smoking, and year quit smoking (if at time of survey the respondent was a former smoker).

Merge NHIS Sample Adult files with MAX Person Summary Files 1999-2008

NHIS Sample Adult file respondents with Medicaid claims in MAX/PS file

**N=38,519**

Apply age exclusion, for each enrollment year

Enrollees aged 18 to 64 years during an enrollment year

**N=28,173**

Apply other exclusions, for each enrollment year

Enrollees aged 18-64 and:

- Not enrolled in managed care plan
- Not dual enrolled in Medicare
- Enrolled in a state with cessation benefits

**N=15,072**

Apply pregnancy benefit only exclusion, for each enrollment year

Enrollees not enrolled because pregnant **N=14,016**

Final analysis dataset

**N=5,982**

**B. Drug Coding**

The presence of a smoking cessation drug claim was assessed in two steps. First, we constructed a table of all smoking cessation drug codes found in the National Drug Code (NDC) database maintained by the Food and Drug Administration (FDA) [1]. National Drug Codes supplied to FDA by the manufacturers are not standardized. Specifically, they are not always the 12 digits required by the MAX files, and many have embedded hyphens. If the NDC was not the expected 12 digits (without hyphens), we expanded it to 12 digits using guidance found on the MAX prescription drug file data dictionary [2], and by comparing the FDA drug codes to lists from the Massachusetts Department of Public Health (Thomas Land, personal communication, September 27, 2012) and from the Wisconsin Department of Health Services [3]. Second, we matched our newly created NDC table of smoking cessation drug codes to the MAX prescription drug file, which contains prescribed drugs, over-the-counter drugs, and other items dispensed by a free-standing pharmacy (non-hospital based).

**References**

1. Food and Drug Administration (FDA). National Drug Code Directory. Available from: http://www.fda.gov/drugs/informationondrugs/ucm142438.htm.
2. Mathematica Policy Research. Medicaid Analytic Extract Drug (RX) Record Layout and Description, 2009. Report submitted to the Centers for Medicare & Medicaid Services, Baltimore, MD. March 31, 2012. Available from: http://www.cdc.gov/nchs/data_access/data_linkage/cms_medicaid.htm.
3. Wisconsin Department of Health Services. ForwardHealth Drug Search Tool. Available from: https://www.forwardhealth.wi.gov/WIPortal/Drug%20Search/tabid/217/Default.aspx.
